# Analysis of Head Movement in KPSIT Dummies and the Impact of Seats and Seat Belts during Low-Speed Collisions 20 km/h

**DOI:** 10.3390/s24175714

**Published:** 2024-09-02

**Authors:** Milos Poliak, Damian Frej, Marek Jaśkiewicz, Jacek Caban, Aleksander Górniak, Mirosław Gidlewski, Iwona Ewa Hajduk, Przemysław Kubiak, Dariusz Tarnapowicz

**Affiliations:** 1 Department of Road and Urban Transport, University of Žilina, 010-26 Žilina, Slovakia; milos.poliak@fpedas.uniza.sk (M.P.); iwona.tomaszewska@gmail.com (I.E.H.); 2 Department of Automotive Engineering and Transport, Kielce University of Technology, Avenue Tysiaclecia Państwa Polskiego 7, 25-314 Kielce, Poland; 3 Faculty of Mechanical Engineering, Lublin University of Technology, Nadbystrzycka 36, 20-618 Lublin, Poland; j.caban@pollub.pl; 4 Department of Automotive Engineering, Mechanical Faculty, Wroclaw University of Science and Technology, 50-370 Wroclaw, Poland; aleksander.gorniak@pwr.edu.pl; 5 Institute of Vehicles and Transportation, Military University of Technology (WAT), gen. Sylwestra Kaliskiego 2 Street, 00-908 Warsaw, Poland; miroslaw.gidlewski@wat.edu.pl; 6 Ecotechnology Team, Lodz University of Technology, 266 Piotrkowska Street, 90-924 Lodz, Poland; przemyslaw.kubiak@p.lodz.pl; 7 Faculty of Mechatronics and Electrical Engineering, Maritime University of Szczecin, Willowa 2, 71-650 Szczecin, Poland; d.tarnapowicz@pm.szczecin.pl

**Keywords:** crash tests, anthropometric dummy, safety

## Abstract

The aim of this study was to compare the head displacement of the KPSIT C50 dummy, representing a 50th percentile male, with the KPSIT C5 dummy, representing a 5th percentile female, during low-speed collisions. Low-speed collisions, such as those occurring in urban traffic jams, are increasingly common. The research was conducted on a dedicated educational workstation designed to measure forces in seat belts. This study is part of a comprehensive research project on crash tests involving both volunteers and physical KPSIT dummies. The tests were conducted at a speed of 20 km/h to simulate real-world low-speed collision scenarios. The findings demonstrate that using a sports bucket seat with four-point or five-point harnesses significantly reduces head displacement compared with standard car seats. Such seating configurations enhance safety by minimizing the risk of head injuries, which can occur when airbags do not deploy during low-speed collisions. Moreover, the study highlights that standard three-point seat belts allow for greater forward head movement, increasing the risk of head contact with the vehicle’s interior during collisions at speeds too low to trigger airbag deployment.

## 1. Introduction

Presently, there is a global initiative led by the European Union and numerous countries worldwide to mitigate the number of fatalities resulting from road accidents. Central to this initiative is the “Vision Zero” philosophy, which has been widely adopted by these nations. The fundamental goal of Vision Zero is to establish and oversee a transportation system in which human error does not lead to fatalities or severe injuries [1,2,3]. Among the most prevalent errors contributing to road accidents are the failure to adapt speed to road conditions and the failure to maintain a safe distance from the vehicle in front [4,5,6].

In line with the Vision Zero philosophy, the management of vehicle safety aims to provide individuals who make errors (such as speeding or failing to maintain distance) with opportunities to prevent accidents or mitigate the severity of injuries through the utilization of active and passive safety systems. Modern automobiles are now equipped with an array of safety features, including obstacle detection, active cruise control, lane-keeping assist, and blind spot detection [7,8,9]. These systems collectively enhance the safety of all road users. However, it is essential to recognize that seat belts constitute the fundamental safety system in every vehicle. Originally introduced into mass production by Ford in 1955 and Volvo in 1958, seat belts were primarily intended to restrain drivers’ and passengers’ bodies during collisions [10,11,12].

At the moment of impact, the force of inertia propels a person’s body forward, increasing the likelihood of collision with various cabin elements or even expulsion through the front windshield. To mitigate such risks, the introduction of airbags has played a pivotal role in reducing injuries to drivers and passengers during crash scenarios. In today’s automotive landscape, it is challenging to conceive of a vehicle devoid of both airbags and seat belts [13,14,15]. Nevertheless, it’s worth acknowledging the significant transformations that motor vehicles have undergone in this era of automotive revolution. Modern vehicles feature adjustable steering columns and customizable car seats, providing enhanced comfort and safety for drivers and passengers alike [16,17,18]. These advancements reflect an ongoing commitment within the automotive industry to prioritize both comfort and safety in vehicle design and manufacturing.

In addition to advancements in vehicle safety features, notable changes have also taken place in road infrastructure. Across many countries, significant expansions of highways and expressways have occurred, often resulting in unidirectional traffic flow. This alteration in road layout has contributed to a reduction in frontal and side collisions observed in recent decades [19,20,21]. However, despite these improvements, rear-end collisions remain a persistent concern, particularly at signalized intersections or during sudden stops in urban traffic. Even at low speeds, such collisions can lead to severe injuries or fatalities. It is crucial for car manufacturers to not overlook the significance of these incidents. At speeds as low as 20 km/h, airbags may not deploy, and standard seat belts may not fully engage due to the relatively minor impact force. Consequently, drivers and passengers risk impacting the vehicle’s dashboard in such scenarios [22,23,24,25]. These realities underscore the ongoing need for advancements in vehicle safety technology, coupled with continued efforts to improve road infrastructure and promote safe driving practices.

Regrettably, it isimportant to acknowledge that each crash test dummy is specifically designed for a particular type of crash test at a predetermined collision speed. While these dummies may accurately simulate the human body’s biofidelity, manufacturers generally do not endorse using a single dummy for multiple crash tests. Moreover, there is currently a scarcity of specialized dummies tailored to low-speed crash simulations available in the market [26,27,28].

In the realm of crash testing, the joints utilized in anthropometric dummies, such as the Hybrid III, require meticulous disassembly, calibration, and reassembly after undergoing a series of collisions [29,30,31]. By contrast, the newly developed KPSIT C50 dummy eliminates the need for recalibration post-collision testing. This highlights a significant advantage of the KPSIT C50 design, offering enhanced convenience and efficiency in crash test procedures [32,33].

It is worth emphasizing that an optimal design solution for anthropometric dummies intended for crash testing would entail a construction that minimizes the need for frequent recalibration while ensuring joint durability and consistent test result reproducibility [28,34,35]. This underscores the importance of ongoing research and innovation in the field of crash test dummy design to improve testing accuracy and reliability.

This article covers the topic of low-speed crash tests. The second chapter presents the research setup for conducting these tests and describes the research methodology. In this context, the tools used, measurement procedures, and data collection guidelines are discussed. The third chapter presents the results of the conducted research. The observed phenomena and collected data on displacement, acceleration, and other parameters of low-speed collisions are described. The influence of various factors, such as the type of car seat or seat belt, on the results of crash tests is also analyzed. Chapter four contains a discussion of the obtained results. The consistency of the collected data with previous studies available in the literature is analyzed. Differences and similarities in the results are compared, with attention to potential factors that may affect the interpretation of the results. Valuable conclusions from these studies can have significant implications for further scientific research and engineering practice related to improving vehicle safety and reducing injuries resulting from collisions.

## 2. Materials and Methods

Research on the movement of body parts, especially the head, during low-speed crash tests was conducted over four years. Initially, the literature on crash testing was analyzed. According to the requirements of dummy manufacturers, each dummy available on the market is tailored to a specific type of crash test (frontal, side, rear) and for testing within a specific collision speed range. Crash tests are most often conducted at speeds from 30 km/h to 64 km/h, and in the case of “whiplash” tests, speeds of 8 km/h or 16 km/h are selected. It should be noted that low-speed collisions at 20 km/h are not commonly conducted by research centers, and there is also a lack of dummies specifically designed for such collisions.

As part of the research, new KPSIT dummies were constructed that correspond to the dimensions and weight of an average man (50th percentile) and a small woman (5th percentile). The 5th percentile dummy simulates smaller individuals, often women, while the 50th percentile dummy represents an average-sized man. This selection allowed for the evaluation of safety mechanisms across a wide spectrum of body types, ensuring the broad applicability of the research results. These dummies were compared with the Hybrid III dummy and the KPSIT C50 simulation model, allowing for an assessment of their behavior during crash tests.

Experimental studies were conducted at the Faculty of Automotive Vehicles and Transport of the Kielce University of Technology using an educational station designed to simulate low-speed collisions and measure forces acting on seat belts. The research station allows for crash tests at speeds ranging from 5 km/h to 25 km/h, enabling rear, side, and frontal crash tests. The experimental setup included two separate measurement circuits. The first circuit recorded the acceleration of the test platform and seat and monitored the forces acting on the seat belts. The second measurement system was specifically designed to document crash tests using a high-speed Digital Phantom V310 camera. This approach enabled a detailed analysis of the dynamic responses of the dummies during simulated collisions, providing valuable insights into their comparative performance and effectiveness in crash scenarios.

Before each series of tests, the dummies were meticulously calibrated to ensure measurement accuracy. Calibration involved checking the dummies’ joints and adjusting them to specific research requirements. Special attention was given to ensuring that the dummies were properly positioned and that their parameters were consistent with real test conditions, which was particularly important when comparing results between different types of dummies.

During the research, the authors encountered several significant challenges. The lack of existing dummies for low-speed tests necessitated the construction of new models that would meet the specifications and research objectives. It was also necessary to ensure that the calibration of the dummies was precise enough for the results to be reliable and suitable for further comparative analyses. Additionally, the complexity of the data analysis required advanced methods to accurately assess the differences in dummy behavior in various crash scenarios. These challenges demanded an innovative approach and the application of advanced engineering techniques, which allowed for obtaining valuable and reliable research results.

**a.**  **RESEARCH GROUP**

**Figure 1:**
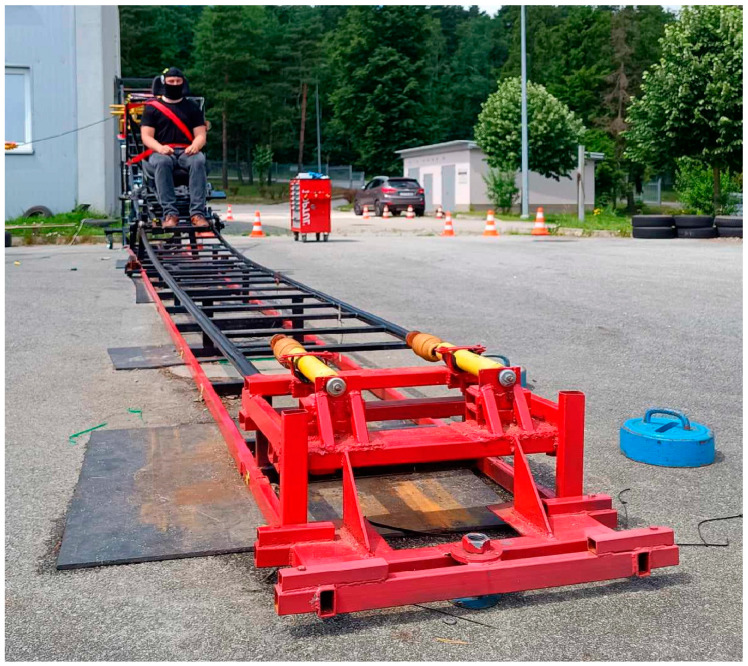
Test stand.

Experimental research was conducted at the Department of Automotive Vehicles and Transport at the Faculty of Mechatronics and Machine Construction, Kielce University of Technology, in Kielce. The research was carried out on an educational workstation designed for simulating low-speed collisions and measuring forces in seat belts. The research workstation is depicted in Figure 1. The workstation enables the execution of low-speed collision tests ranging from 5 km/h to 25 km/h. The research setup features a measurement track with a length of 10 m along which a test platform with a car seat can freely move. The workstation allows for conducting rear, side, and frontal collision tests. The collision tests were conducted outdoors on a sunny day. The collision tests were recorded using a high-speed Digital Pantom V310 camera.

**b.**  **IMPLEMENTATION OF EXPERIMENTAL RESEARCH**

**Figure 2:**
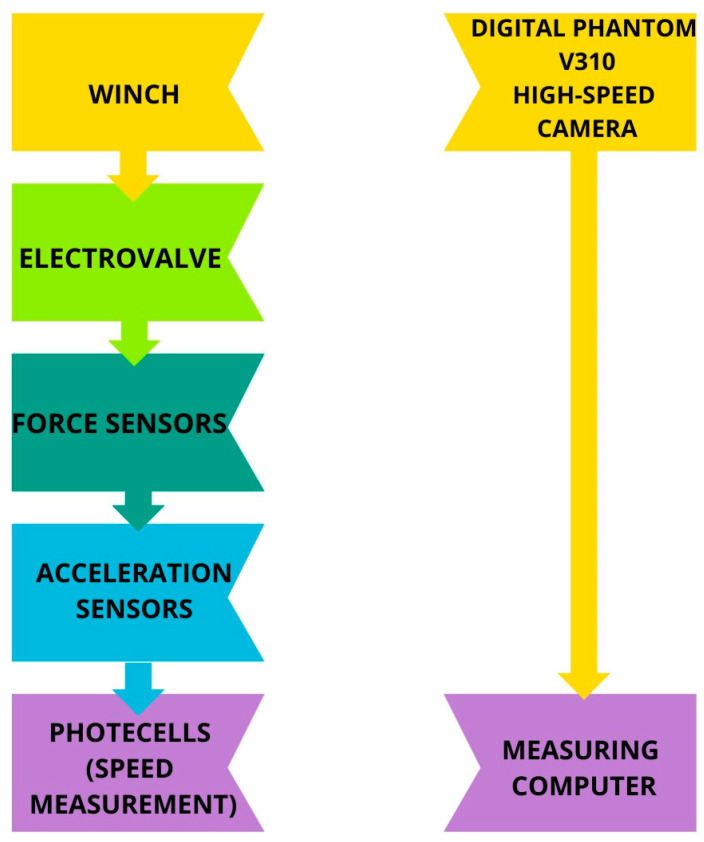
Diagram of the stand used for crash tests.

The study aimed to investigate and compare head displacement between the KPSIT C50 and KPSIT C5 dummies in a simulated crash scenario. Initially, crash simulations were performed employing the KPSIT C50 dummy, succeeded by tests with the KPSIT C5 dummy. The experimental setup comprised two distinct measurement circuits. The first circuit captured the acceleration of the research platform and the tested seat, along with monitoring the forces exerted on the seat belts. Meanwhile, the second measurement system was specifically designed to document the crash test using a high-speed Digital Phantom V310 camera. This comprehensive approach allowed for a detailed analysis of the dynamic responses of the dummies during the simulated collisions, providing valuable insights into their comparative performance and effectiveness in crash scenarios. A diagram of the stand used for the crash tests is shown in Figure 2.

In the experimental low-speed crash tests at 20 km/h, the precise control of variables such as the seat adjustment angle and seatbelt tension was crucial to ensure the reliability of the results. Before each test, the seatback angle was precisely measured to ensure uniform test conditions. An inclinometer, a specialized device known for its high accuracy in measuring tilt angles, was used for this purpose. The inclinometer allowed for the precise adjustment of the seat to a specific angle, eliminating discrepancies due to inconsistent seating positions in different tests.

To ensure proper seatbelt tension, thorough inspections were conducted before each test. The belts needed to be adequately tightened to eliminate any slack between them and the dummy’s body, which is key to replicating real-world collision conditions. Simultaneously, the tension had to be high enough to prevent body displacement but not cause discomfort. The target was to achieve a belt tension force of 50 N. Precise tension measurements were conducted using a handheld dynamometer, allowing for the accurate determination and maintenance of consistent belt tension levels in each test.

The application of these procedures ensured high consistency and repeatability of the experiments, contributing to the acquisition of reliable data on the head movement of KPSIT dummies during low-speed collisions. This approach ensured that the study results were robust and could serve as a basis for further analyses of the impact of seatbelts and seats on passenger safety.

**c.**  **DATA ANALYSIS**

**Figure 3:**
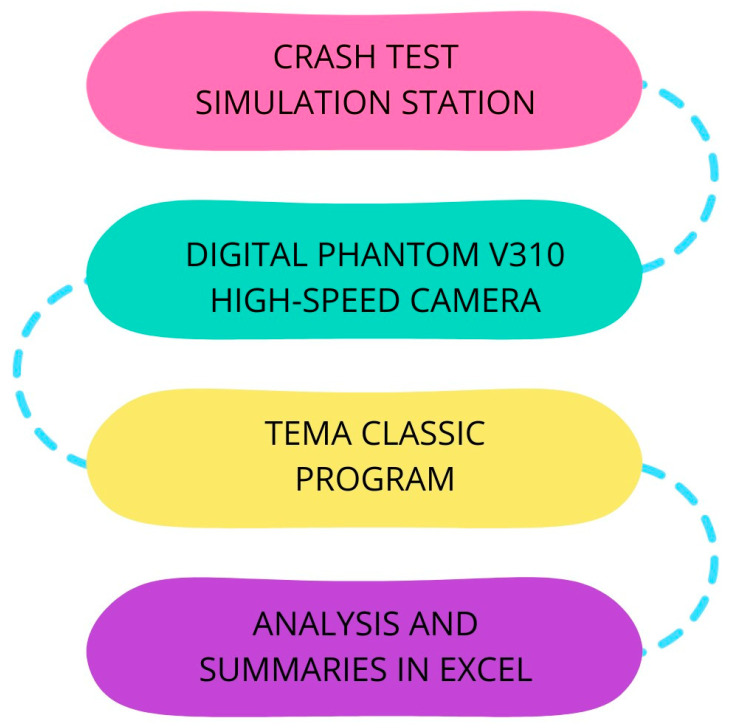
Scheme of measurement data recording on a crash test stand.

The schematic representation of the data acquisition process is depicted in Figure 3. Both the KPSIT C50 and KPSIT C5 dummies underwent crash tests at identical collision speeds. The captured crash test footage from the Digital Phantom V310 camera was initially analyzed using TEMA CLASSIC T2020c software. TEMA CLASSIC is an advanced motion analysis software used in crash tests to precisely track the trajectory and velocity of objects, such as test dummies, using video recordings. It allows you to visualize and calculate speed and acceleration to help you evaluate the effectiveness of vehicle safety systems.

Following this, the data pertaining to the displacements of specific body parts of the dummies were transcribed into an Excel 2019 spreadsheet. This facilitated the creation of graphs illustrating the comparative displacements of different components of the dummies under test conditions. This meticulous data processing approach enabled a comprehensive examination of the performance differences between the KPSIT C50 and KPSIT C5 dummies during crash simulations.

**d.**  **Object of experimental research**

**Figure 4:**
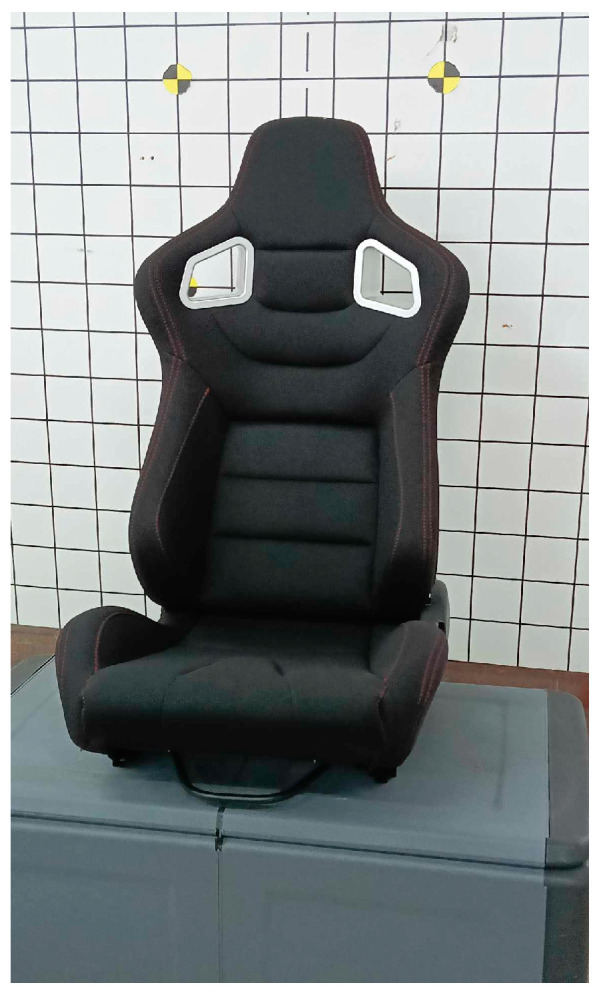
Sport bucket car seat.

**Figure 5:**
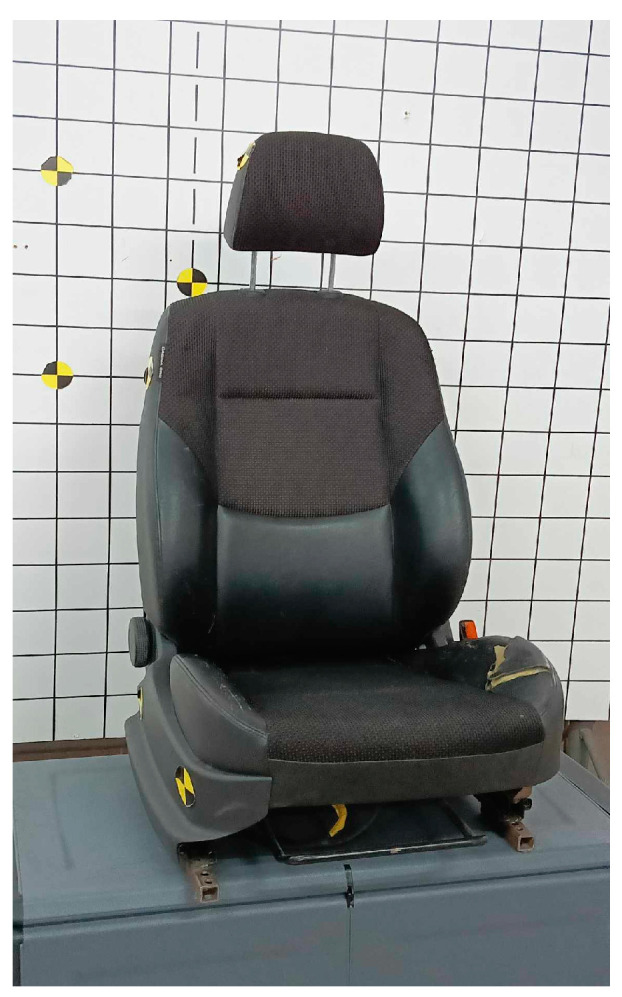
Passenger car seat.

The crash tests of the KPSIT C50 and C5 dummies involved frontal, rear, and side collisions. Each crash test using the dummy was repeated 5 times to ensure the credibility and repeatability of the data. The final analysis comparing the displacement of the dummy’s head was conducted based on averaged data from the 5 samples. The crash tests were conducted using two types of car seats, as depicted in Figure 4 and Figure 5. The recline angle of the seat back relative to the ground during the crash tests was 110°, and this range was checked and corrected after each series of 5 measurements. Tests using the bucket seat were conducted with four-point and five-point harnesses, while tests using the regular car seat were conducted with three-point and four-point harnesses.

The aim of the article was to determine the differences in head displacement between the KPSIT C50 and KPSIT C5 dummies during a collision test at a speed of 20 km/h. This speed is particularly significant because it represents low-speed impacts, which are often overlooked in traditional crash tests but are frequent in urban traffic scenarios. Understanding the dynamics at this speed helps improve safety measures for everyday driving conditions.

The selection of two car seats aimed at examining the differences in the trajectory of the dummy’s head movement, which provides insights into how seat design can impact occupant safety during collisions. A sports bucket seat significantly differs in construction from standard car seats, offering enhanced lateral support and a snugger fit. These seats are specifically designed to be better tailored to the human body, providing increased stability during vehicle operation, which can be crucial in high-performance and racing environments where sharp maneuvers and high g-forces are common.

Additionally, the use of four-point and five-point harnesses in the study highlights the importance of advanced restraint systems. These harnesses reduce the possibility of lateral body movement and stabilize the human posture during driving, unlike standard seat belts, which do not maintain the body posture in one position during vehicle operation. Standard seat belts allow for some freedom of movement and include features like automatic belt length adjustment to accommodate different occupant sizes and positions. However, in the context of collision safety, particularly during low-speed impacts, the additional restraint provided by multi-point harnesses can significantly enhance occupant protection by minimizing the risk of injuries due to sudden lateral or forward movement.

This research underscores the necessity of evaluating seat and restraint designs in various crash scenarios to optimize safety features that can mitigate injury risks across different impact speeds and angles. By focusing on both the type of seat and the restraint system, this study provides valuable data that can inform future automotive safety designs and regulations, ultimately aiming to enhance the overall safety of vehicle occupants in real-world conditions.

## 3. Results of Experimental Studies

**Figure 6:**
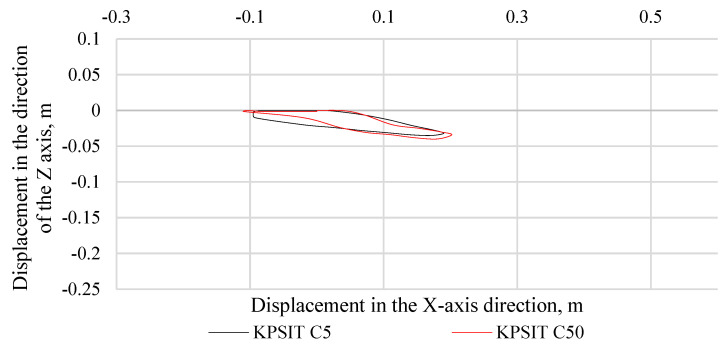
Head trajectory of KPSIT dummies during a frontal crash test at 20 km/h—four-point belts, sports bucket seat.

**Figure 7:**
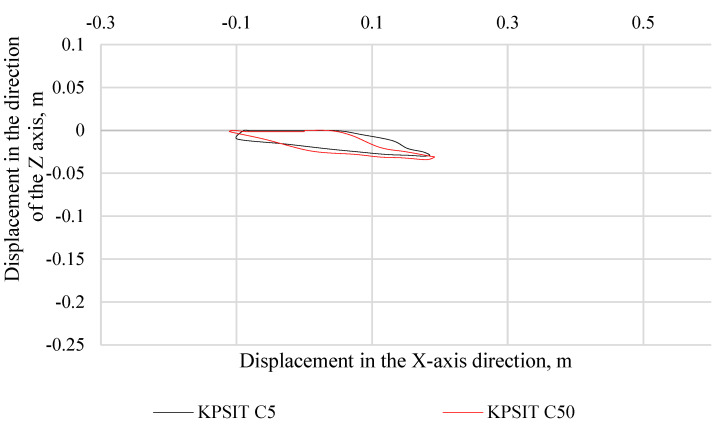
Head trajectory of KPSIT dummies during a frontal crash test at 20 km/h—five-point belts, sports bucket seat.

In Figure 6, the trajectory of the KPSIT C50 and KPSIT C5 dummy heads during a frontal collision test at a speed of 20 km/h is depicted, using four-point safety harnesses and a sports bucket seat. In Figure 7, the trajectory of the KPSIT C50 and KPSIT C5 dummy heads during a frontal collision test at a speed of 20 km/h is shown, using five-point safety harnesses and a sports bucket seat. When four-point harnesses were employed, the maximum head displacement in the *X*-axis direction was recorded for the KPSIT C50 dummy, amounting to 0.20 m. The differences in head trajectory values during the collision test between the KPSIT C50 and KPSIT C5 dummies were minor, not exceeding 15%. This difference was calculated on the basis of the sum of differences in each point of the head trajectory within a specified time interval (from 0.00 s to 0.40 s), then averaged and presented as a percentage value. When five-point harnesses were used, the maximum head displacement in the *X*-axis direction was recorded for the KPSIT C50 dummy, amounting to 0.195 m. The differences in head trajectory values during the collision test between the KPSIT C50 and KPSIT C5 dummies were negligible, not exceeding 12%.

**Figure 8:**
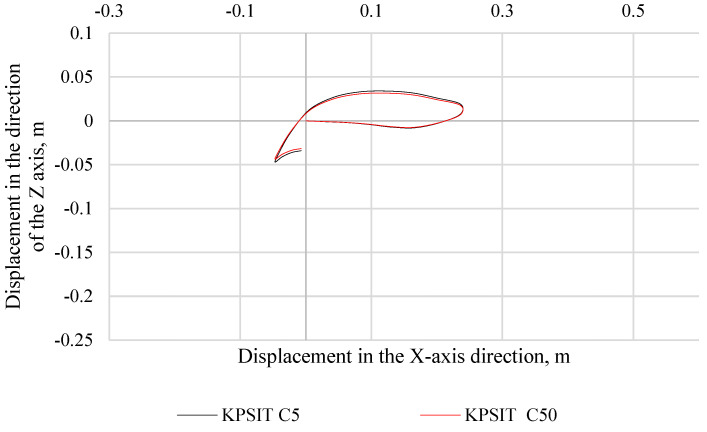
Head trajectory of KPSIT dummies during a rear-end crash test at 20 km/h—four-point belts.

**Figure 9:**
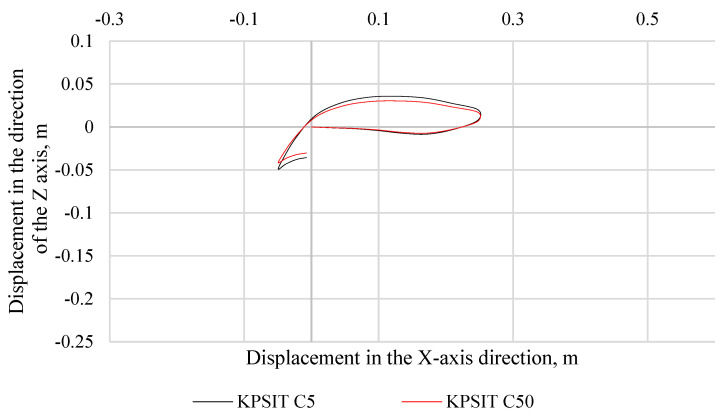
Head trajectory of KPSIT dummies during a rear crash test at 20 km/h—five-point belts.

In Figure 8, the trajectory of the KPSIT C50 and KPSIT C5 dummy heads during a rear collision test at a speed of 20 km/h is presented, using four-point safety harnesses and a sports bucket seat. In Figure 9, the trajectory of the KPSIT C50 and KPSIT C5 dummy heads during a rear collision test at a speed of 20 km/h is depicted, using five-point safety harnesses and a sports bucket seat.

When four-point harnesses were used, the maximum head displacement in the *X*-axis direction was recorded for the KPSIT C50 dummy, amounting to 0.235 m. The differences in head trajectory values during the collision test between the KPSIT C50 and KPSIT C5 dummies were minor, not exceeding 8%.

When five-point harnesses were used, the maximum head displacement in the *X*-axis direction was recorded for the KPSIT C50 dummy, amounting to 0.25 m. The differences in head trajectory values during the collision test between the KPSIT C50 and KPSIT C5 dummies were negligible, not exceeding 10%.

**Figure 10:**
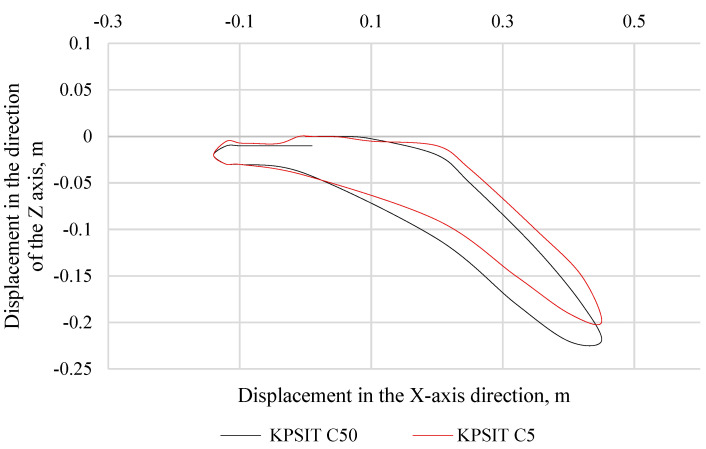
Head trajectory of KPSIT dummies during a frontal crash test at 20 km/h—three-point belts.

In Figure 10, the trajectory of the KPSIT C50 and KPSIT C5 dummy heads during a frontal collision test at a speed of 20 km/h is presented, using three-point safety belts and a regular car seat. When three-point seat belts were used, the differences in head trajectory values during the collision test between the KPSIT C50 and KPSIT C5 dummies were minor, not exceeding 12%.

**Figure 11:**
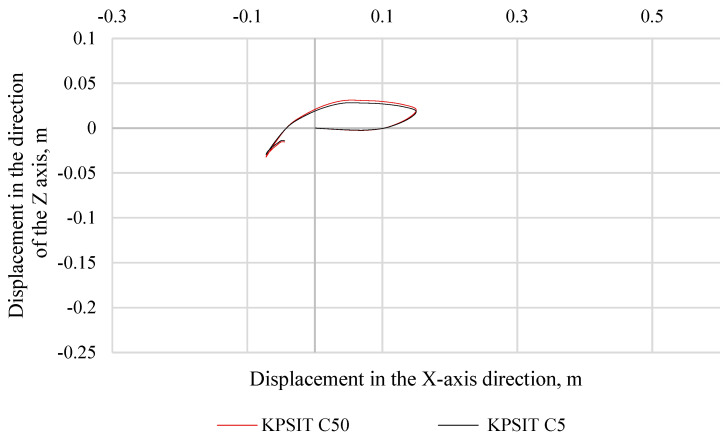
Head trajectory of KPSIT dummies during a rear crash test at 20 km/h—three-point belts.

In Figure 11, the trajectory of the KPSIT C50 and KPSIT C5 dummy heads during a rear collision test at a speed of 20 km/h is depicted, using three-point safety belts and a regular car seat. When three-point seat belts were used, the differences in head trajectory values during the collision test between the KPSIT C50 and KPSIT C5 dummies were minor, not exceeding 10%.

**Figure 12:**
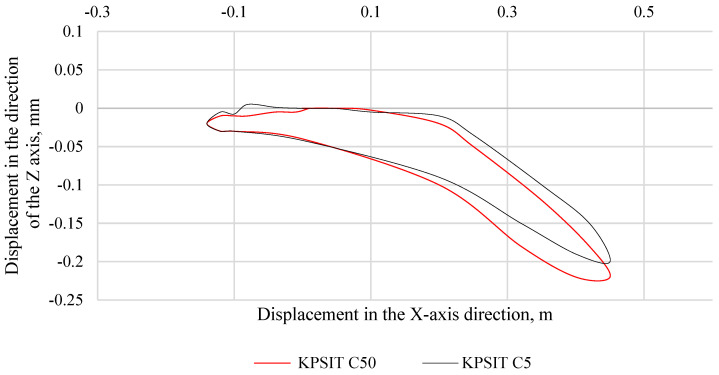
Head trajectory of KPSIT dummies during a frontal crash test at 20 km/h—four-point belts.

In Figure 12, the trajectory of the KPSIT C50 and KPSIT C5 dummy heads during a frontal collision test at a speed of 20 km/h is shown, using four-point safety belts. When four-point seat belts were used, the differences in head trajectory values during the collision test between the KPSIT C50 and KPSIT C5 dummies were minor, not exceeding 10%.

**Figure 13:**
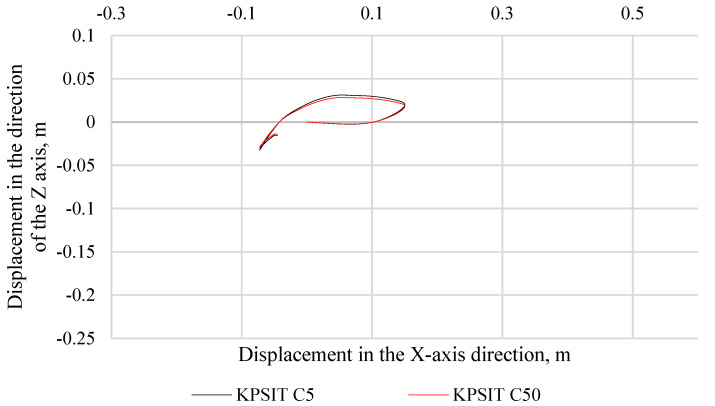
Head trajectory of KPSIT dummies during a rear-end crash test at 20 km/h—four-point belts.

In Figure 13, the trajectory of the KPSIT C50 and KPSIT C5 dummy heads during a rear collision test at a speed of 20 km/h is depicted, using four-point safety belts. When four-point seat belts were used, the differences in head trajectory values during the collision test between the KPSIT C50 and KPSIT C5 dummies were minor, not exceeding 8%.

**Figure 14:**
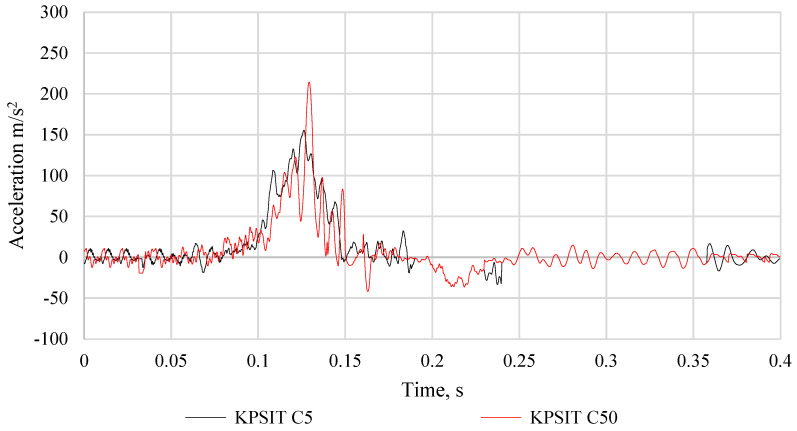
Comparison of the head acceleration of the KPSIT C50 dummy and the KPSIT C5 dummy during the frontal crash test at 20 km/h—four-point belts, sports bucket seat.

The comparison of the head acceleration of KPSIT C50 and KPSIT C5 dummies during a frontal crash test at a speed of 20 km/h, using four-point safety harnesses and a sports bucket seat, is presented in Figure 14. It should be noted that during the collision, the maximum head acceleration value was recorded as 205 m/s^2^ for the KPSIT C50 dummy and 152 m/s^2^ for the KPSIT C5 dummy. Lower values were observed for the KPSIT C5 dummy during the collision when using four-point safety harnesses and a sports bucket seat.

**Figure 15:**
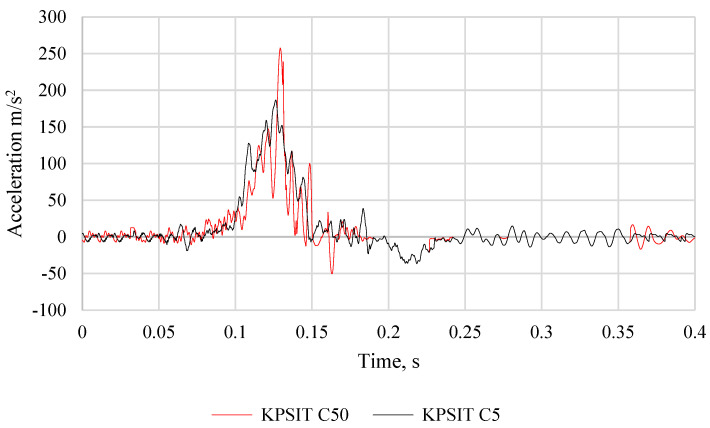
Comparison of the head acceleration of the KPSIT C50 dummy and the KPSIT C5 dummy during a frontal crash test at 20 km/h—five-point belts, bucket sports seat.

The comparison of head acceleration between the KPSIT C50 and KPSIT C5 dummies during a frontal crash test at a speed of 20 km/h using a five-point safety harness and a sports bucket seat is presented in Figure 15. It should be noted that during the collision, the maximum head acceleration value was recorded as 255 m/s^2^ for the KPSIT C50 dummy and 173 m/s^2^ for the KPSIT C5 dummy. Lower values were observed for the KPSIT C5 dummy during the collision when using five-point safety harnesses and a sports bucket seat.

**Figure 16:**
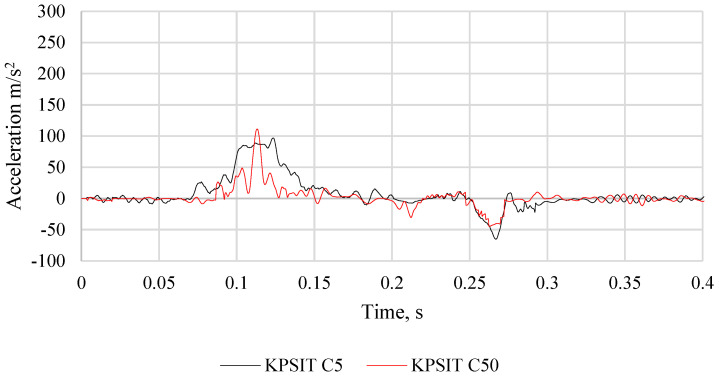
Comparison of the head acceleration of the KPSIT C50 dummy and the KPSIT C5 dummy during a frontal crash test at 20 km/h—three-point belts passenger car seat.

The comparison of head acceleration between the KPSIT C50 and KPSIT C5 dummies during a frontal crash test at a speed of 20 km/h using three-point safety belts and a passenger car seat is depicted in Figure 16. It should be noted that during the collision, the maximum head acceleration value was recorded as 110 m/s^2^ for the KPSIT C50 dummy and 100 m/s^2^ for the KPSIT C5 dummy. Lower values were observed for the KPSIT C5 dummy during the collision when using three-point safety belts and a passenger car seat.

**Figure 17:**
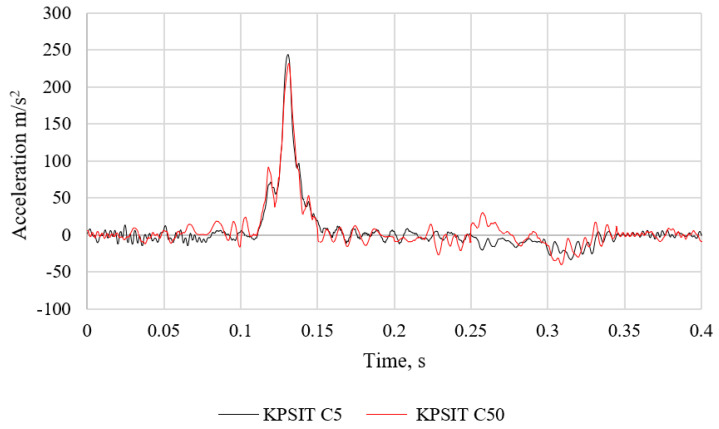
Comparison of acceleration of KPSIT C50 dummy and KPSIT C5 dummy during rear crash test at 20 km/h—three-point belts, seat, passenger car.

The comparison of acceleration between the KPSIT C50 and KPSIT C5 dummies during a rear-end crash test at a speed of 20 km/h using three-point safety belts and a passenger car seat is depicted in Figure 17. It should be noted that during the collision, the maximum head acceleration value was recorded as 240 m/s^2^ for the KPSIT C50 dummy and 245 m/s^2^ for the KPSIT C5 dummy. Lower values were observed for the KPSIT C50 dummy during the collision when using three-point safety belts and a passenger car seat.

## 4. Discussion

Article [34] describes a research setup and a specialized control system with data acquisition for experimental analysis of physical parameters during vehicle crash tests. These tests were conducted at low speeds using a dummy carriage, employing various types of seat belts ranging from two-point to multi-point configurations. Details regarding the research setup, control system, and measurement apparatus utilized are outlined. During the experiments, the authors delineated the force profiles acting on the three-point section of the lap and shoulder belts within collision speeds ranging from 15 km/h to 17 km/h. The authors demonstrated that despite the low speeds, the force values between the dummy and the three-point seat belts can reach significant magnitudes, even up to 5 kN, with a dynamic increase on the order of 30 kN/s. In the ongoing study, a modified research setup was employed for crash tests. The setup was expanded, and the measurement track was extended from 7 m to 10 m. The final 3 m of the measurement track were horizontally positioned relative to the ground to stabilize the collision velocity.

Previous articles [32,33] have focused on comparing the results of crash tests conducted on volunteers with computer simulation using a dummy. The authors conducted a series of crash tests on volunteers, followed by a simulation using a dummy in the MSC ADAMS 2023 program. The simulation results of dummy head displacement aligned with the results observed during volunteer crash tests, validating the credibility of the simulation model crafted by the authors in the MSC ADAMS 2023 program. In the ongoing study, a series of five crash tests were conducted under identical conditions. The results from the crash test series were averaged to reduce errors stemming from individual tests.

One article [35] presented a research setup and a control and data acquisition system for experimental low-speed crash tests. The authors conducted experimental crash tests involving volunteers. The authors’ studies encompassed volunteers of both genders, and the results indicated that the trajectory of female volunteers’ heads aligned with that of male volunteers. These findings suggest that the construction of an anthropometric dummy for low-speed crash tests can be based on data averaged across the entire population, without the need to account for gender differences. In the ongoing study, it was demonstrated that the difference in head displacement between KPSIT C50 and KPSIT C5 dummies ranged from 8% to 12%. The results suggest that differences in the mass and dimensions of individual dummy body parts during low-speed crash tests do not induce changes. Perhaps one KPSIT dummy representing the 50th percentile population is sufficient for determining displacements, irrespective of gender.

Another article [36] conducted research aimed at examining the acceleration and force resulting from contact between go-karts, a popular amusement park attraction. During 44 low-speed collisions between a stationary go-kart and a moving go-kart involving passengers, one of the measuring devices used was a male 50th percentile Hybrid III ATD. The authors demonstrated that the acceleration and velocity changes of the go-karts increased with higher collision speeds, while the collision duration and restitution decreased at higher collision speeds. Injury indices such as HIC remained below harmful thresholds. In the ongoing study, maximum head acceleration during collisions, ranging from 100 to 260 m/s^2^, was recorded, depending on the type of car seat and seat belt used. The lowest acceleration values occurred when standard three-point belts were employed. Unfortunately, when using these belts, the dummy head displacement was several times greater than when using five-point or four-point belts.

The research conducted in [37] showed that tests at a speed of 56 km/h do not favor the use of technologies reducing chest injuries in cases of lower force. The authors analyzed the benefits of conducting crash tests with a lower-force front barrier (40 km/h) using female dummies restrained by 5% belts and more stringent requirements regarding chest injury. The authors’ preliminary analysis suggested that a standard limiting chest deflection to 34 mm could reduce the risk of serious chest injuries by 16–24% in the case of seat-belted individuals during frontal collisions. In the ongoing study, chest deflection was not measured. The authors focused solely on dummy head displacement and acceleration during crash testing. Head displacement on the order of 0.3 m can cause the head to strike the vehicle’s cockpit. Collision at such a speed does not always activate the airbag. Therefore, improperly positioned car seats may cause additional injuries to the occupant when the head strikes the car’s cockpit during a low-speed collision. Unfortunately, such collisions are often encountered in urban traffic jams.

In another article [38], the authors evaluated the effectiveness of Autonomous Emergency Braking (AEB) technology at low speeds in current passenger vehicle models, based on real accident experiences. The authors’ research results showed an overall reduction in rear-end collision accidents by 38% for vehicles equipped with AEB compared to a control group of similar vehicles. Additionally, the authors found no statistical evidence of any difference in the effectiveness of AEB between urban areas (≤60 km/h) and rural areas (>60 km/h). The research in [39] suggests that current crash test dummies are based on European and American models, which may lead to limitations in predicting injuries in other regions. The authors demonstrated that further research is needed on crash test dummies that take into account differences between countries and develop dummies representing different groups of people, such as older individuals or those with obesity. In the ongoing study, KPSIT dummies were used; these are dedicated to low-speed crash tests in which the most important parameter is the displacement and acceleration of individual body parts at the moment of impact.

The aim of the study in [40] was to examine changes in the frequency, risk, and patterns of lower limb injuries depending on vehicle and passenger parameters as a function of the vehicle model year. To achieve this, the authors analyzed 10,988 observations representing 4.7 million drivers involved in frontal collisions. The authors applied a logistic regression model to understand the relationship between persistent lower limb injuries and characteristics of car accidents, such as vehicle type, model year, and the intrusion of footwells and dashboards, as well as passenger characteristics like age, gender, height, and weight. The authors’ research showed that the risk of lower limb injuries in women was higher than in men. According to the authors, increased driver mass had a positive relationship with injury occurrence, while age and height did not affect the likelihood of injury. In the ongoing study, the authors confirmed that the type of seat belt and car seat had an impact on injuries during low-speed accidents. The authors emphasized that a properly selected and adjusted car seat contributes to reducing the risk of injury during low-speed collisions.

In [41], the authors highlighted a significant difference in the number of fatalities and injuries in car accidents between male and female drivers. Female drivers are 13% more likely to die in similar car accidents compared to male drivers. Meanwhile, in [42], the authors presented the full results of a low-intensity frontal collision in which a small vehicle struck a stationary vehicle at a speed of about 12 m/s, causing it to overturn. The authors’ findings indicated that even a low-intensity collision poses a significant threat and carries the risk of vehicle rollover as well as head and neck injuries to the passenger. Even at a relatively low speed, the driver experienced minor neck and head injuries in the following days, and the longitudinal damages were significant. In the ongoing study, the authors confirmed that despite the low collision speed, head acceleration in the range of 100 to 260 m/s^2^ can cause serious cervical injuries.

## 5. Conclusions

In the literature on the subject, a definition of low-speed collision up to 25 km/h has been adopted. For low-speed collisions, rear-end crash tests of the whiplash type are conducted. These are most commonly performed at speeds of 8 km/h and 16 km/h. Low-speed collisions are also conducted in the case of vehicle front bumper tests. These tests are typically carried out at speeds of 5 km/h and 10 km/h. For the experimental crash tests conducted, a speed of 20 km/h was adopted. Both frontal and rear crash tests were performed. On the basis of the crash tests involving KPSIT dummies, the trajectory of head movement was determined. The level of potential bodily injury was assessed on the basis of the displacement of the dummies’ head movement. It should be noted that in the case of frontal collisions, a collision at 20 km/h is not fatal, but due to incorrect seat positioning and improperly fastened seat belts, the accident participant may additionally hit the vehicle’s dashboard, which can cause cervical spine injuries. Moreover, the rebounding head in a frontal collision, striking a poorly adjusted headrest, can further exacerbate cervical spine injuries. In the case of rear collisions at a speed of 20 km/h, it can cause severe cervical spine injuries. Therefore, due to safety reasons, it is not possible to use volunteers in such crash tests.

In the assessment of low-speed collisions, the accuracy and durability of the dummy are of particular importance. The constructed KPSIT dummies can be used for crash tests, including side, rear, and frontal collisions at low speeds. Using a single dummy allows for a comprehensive understanding of the displacements of various body parts at the moment of impact at low speeds. According to traffic accident statistics, the number of frontal and side collisions has significantly decreased in the past decade. This is due to the development of road infrastructure, especially expressways and highways, on which all vehicles move in the same direction. Urban infrastructure has also undergone significant changes, with parallel intersections increasingly being replaced by roundabouts, which also reduce the likelihood of frontal accidents. It can only be observed that rear-end collisions have remained almost at the same level for several years across the EU. Rear-end collisions most often occur at intersections or in traffic jams due to a failure to stop in time. Rear-end collisions are the most dangerous for passengers and the driver of the vehicle because even at low speeds, they can cause serious upper cervical injuries. Therefore, experimental studies of low-speed crash tests are necessary to examine how vehicle elements such as the driver’s seat or seat belts affect body displacement.

The results of experimental studies highlight minimal differences in the head displacement profiles of the KPSIT dummies. It is noteworthy that gender seems to have a negligible impact on the differences observed in the displacement patterns of the dummies’ body parts during low-speed collision simulations. The custom-made KPSIT dummy models tailored for low-speed impact simulations prove to be essential tools for uncovering the complexities of human injury mechanisms during collisions. Comparative analyses of the head displacement profiles of the dummies reveal a noticeable influence of changes in car seat designs and types of seat belts on the dynamics of head displacement during low-speed collisions. The use of four-point or five-point seat belt configurations ensures less head displacement of the KPSIT C50 dummy during 20 km/h collisions compared to conventional seat belt systems.

Summarizing the comparative assessment of crash test outcomes between KPSIT C50 and KPSIT C5 dummy variants, nuanced differences in head displacement and acceleration profiles surfaced. While both dummy iterations exhibit similar behavioral patterns across frontal, rear, and side impact scenarios at 20 km/h, the KPSIT C50 dummy demonstrates marginally reduced displacement and acceleration metrics relative to its KPSIT C5 counterpart. The implementation of four-point and five-point seatbelt systems within bucket sports seats, alongside three-point seatbelts in passenger car settings, collectively contributes to the mitigation of head displacement for both dummy variants. Conversely, trials featuring three-point seatbelts in passenger car configurations manifest the least accelerative forces, indicative of the efficacy of standard safety mechanisms in passenger vehicles. In essence, while dissimilarities in dummy responses during crash simulations are discernible, overarching parallels underscore the suitability of both dummy models for vehicular safety assessments. Moreover, these nuances furnish pivotal insights for advancing research endeavors aimed at enhancing road transport safety, particularly in the realm of passenger protection system design.

The results of the experimental studies confirmed that gender is not a significant parameter in low-speed crash tests. The differences between the head displacement of the KPSIT C50 dummy and the KPSIT C5 dummy were within 10%. However, it should be noted that the seating preferences of men and women are likely different. Therefore, in further research, the authors will compare the seat settings, including the angle of the backrest and headrest, among volunteers divided by gender.

## Data Availability

The data presented in this study are available on request from the corresponding author.

## References

[1] Almqvist S., Djurberg P., Edgren T., Fredriksson R. (2017). The Development of a New Test Method to Evaluate Pedestrian Head Impact Protection Using an Anthropomorphic Test Device. Traffic Inj. Prev..

[2] Anund A., Fors C., Nordström T. (2020). The Use of the Vision Zero Strategy for Road Safety in Sweden: Investigating the Effect of Increasingly Strict Speed Limits. Accid. Anal. Prev..

[3] Aydin N., Karaca F., Demirci E. (2018). Real-Time Traffic Accident Detection and Condition Analysis Using High-Resolution Traffic Cameras. Transp. Res. Part C Emerg. Technol..

[4] Bach P., Sadagopan A., Morando A., Schoettle B., Gao Y., Al-Naami B., Sherony R., Toma S., Fasil H., Flannagan C. (2020). Vehicle Automation and Electrification on Urban Traffic Energy Consumption: Evidence from Real-World Applications and Implications for Energy Policy. Appl. Energy.

[5] Baumann J., Durbin D.R., Arbogast K.B., Seacrist T. (2014). Comparison of Thoracic Responses of ATD and Pediatric PMHS in Low-Speed Frontal Impacts. Stapp Car Crash J..

[6] Bellem H., Thiel B., Schrauf M., Krems J.F. (2018). Comfort in Automated Driving: An Analysis of Preferences for Different Automated Driving Styles and Their Dependence on Personality Traits. Transp. Res. Part F Traffic Psychol. Behav..

[7] Boström O., Bohman K., Pipkorn B., Jakobsson L., Haland Y., Eriksson J.L., Pywell J., Wolf H., Schell H. (2020). Development and Evaluation of a New Rear-Seat Frontal Impact Concept. Traffic Inj. Prev..

[8] Broberg T., Rosander M., Andréasson R., Pettersson H. (2021). Assessment of Driver Inattention—Two Different Ways of Measuring Driver’s Gaze Behavior. Accid. Anal. Prev..

[9] Brumbelow M.L., Zuby D.S. (2019). Impact of Rear Seat Belt Reminders on Adult Rear Seat Belt Use. Accid. Anal. Prev..

[10] Burns A., Kanarachos S., Thite A., Mandal M., Mathai S.K. (2019). Development of a Framework for the Evaluation of Integrated Safety Systems for Vehicles. J. Saf. Res..

[11] Carlsson A., Bohman K., Jakobsson L., Svensson M.Y. (2021). Performance of Different Rear Seat Frontal Impact Protection Principles for the Adult Passenger. Accid. Anal. Prev..

[12] Chockalingam V., Chinnadurai V., Periyasamy P. (2018). Analysis of Vehicle Dynamics and Stability Control Systems Using the MATLAB/SIMULINK Framework. Transp. Res. Part C Emerg. Technol..

[13] Choi E., Eluru N., Paleti R., Bhat C.R. (2015). Modeling Crash Propensity Using Multilevel Mixed Models: An Analysis of the Effects of Vehicle, Driver, and Roadway Characteristics on Crash Occurrence. Accid. Anal. Prev..

[14] Collins L.M., Kahlon S., McCarthy S., Thompson J. (2020). An Analysis of the Effectiveness of Advanced Driver Assistance Systems (ADAS) in Reducing Road Traffic Crashes: A Case Study of the Australian Market. Accid. Anal. Prev..

[15] Cordell S., Williamson B., Dumitru G., Lim C., Kang H., Shin Y., McAlpine A., Howard D. (2021). Acoustic Signature Prediction of Motor Vehicles in an Urban Environment for Enhanced Pedestrian Safety. Transp. Res. Part D Transp. Environ..

[16] Decker M., McKinley T., Stitz M., Lowry J. (2020). Advancements in Adaptive Cruise Control Systems and Their Role in Enhancing Vehicle Safety. Procedia Comput. Sci..

[17] Dingus T.A., Guo F., Lee S., Antin J.F., Perez M., Buchanan-King M., Hankey J. (2016). Driver Crash Risk Factors and Prevalence Evaluation Using Naturalistic Driving Data. Proc. Natl. Acad. Sci. USA.

[18] Donaldson L., Cohen J., Turoff S., Salunkhe A., Mancuso R., Rubin D. (2021). Integration of Passive and Active Safety Systems in Modern Vehicles: A Review of Current Technologies and Their Effectiveness. IEEE Trans. Intell. Transp. Syst..

[19] Engelbrecht R., Richardson B., Streff F. (2019). Safety Impact of Connected and Automated Vehicle Systems on Traffic Accidents in Michigan. Transp. Res. Part A Policy Pract..

[20] Fagerlind H., Ydenius A., Kullgren A., Ohlin M., Tingvall C. (2020). Driver Behavior in Relation to Automatic Emergency Braking System Activation. Accid. Anal. Prev..

[21] Fujita S., Taniguchi K., Takeuchi M. (2019). The Effects of Infrastructure Development on Traffic Safety: A Study of Expressway Expansion in Japan. J. Transp. Land Use.

[22] Grembek O., Shaheen S. (2020). The Role of Advanced Vehicle Technologies in Achieving Vision Zero Goals: A Case Study of Automated Vehicle Deployment in Urban Areas. J. Saf. Res..

[23] Gribble J.N., Ezzati A.M., Kononov J., Sokolow J. (2021). Effects of Driver Assistance Systems on Traffic Safety in the United States. Transp. Res. Part F Traffic Psychol. Behav..

[24] Hamdar S.H., Mahmassani H.S., Chen R. (2015). Aggressiveness Propensity Index for Driver Behavior Interventions. Accid. Anal. Prev..

[25] Han Y., Zhu X., Wang L., Song C., Lu Y. (2019). Analysis of Front Crash Prevention Technology’s Impact on Reducing Vehicle Accident Rates. Int. J. Automot. Technol..

[26] Henning M.J., Dickerson C.R., McGowan D., Reimer B., Mehler B. (2019). Influence of In-Vehicle Technology on Driver Vigilance and Response Time. Transp. Res. Part F Traffic Psychol. Behav..

[27] Huang Y., Zhang L., Xie L. (2018). Advancements in Airbag Systems for Enhanced Occupant Safety in Side-Impact Collisions. Int. J. Automot. Technol..

[28] Inomata Y., Nakamura M., Tanaka K. (2020). Innovative Approaches to Reduce Rear-End Collisions Using Machine Learning Models in Traffic Flow Analysis. J. Intell. Transp. Syst..

[29] Jia X., Ding Y., Hu Z., Liu H., Feng Z. (2021). Research on the Comprehensive Evaluation of Intelligent Vehicle Safety Systems. IEEE Access.

[30] Johansen T., Lundberg P., Kristensen N. (2020). Effects of Autonomous Vehicles on Road Safety and Traffic Congestion. J. Transp. Saf. Secur..

[31] Frej D. (2024). The Effect of Changing the Angle of the Passenger Car Seat Backrest on the Head Trajectories of the 50th Percentile Male Dummy. Sensors.

[32] Jaśkiewicz M., Frej D., Matej J., Chaba R. (2021). Analysis of the Head of a Simulation Crash Test Dummy with Speed Motion. Energies.

[33] Laureshyn A., Jenvald J., Jonsson T. (2017). Using Video Analysis for Traffic Safety Research: A Systematic Literature Review. Transp. Res. Part F Traffic Psychol. Behav..

[34] Li Y., Yin S., Liu H., Zhao F., Yu Y. (2020). The Effects of Connected Vehicle Technology on Traffic Safety: An Assessment Using Simulation and Real-World Data. IEEE Trans. Intell. Transp. Syst..

[35] Ma T., Wei S., Shao C., Yang D. (2020). Evaluation of the Impact of Advanced Vehicle Safety Technologies on Road Traffic Safety in China. J. Saf. Res..

[36] Kloppenborg N., Amenson T., Wernik J., Wiechel J. (2018). Low-Speed Go-Kart Crash Tests and a Comparison to Activities of Daily Living. ASME J. Risk Uncertain. Part B.

[37] Digges K., Dalmotas D. (2007). Benefits of a Low Severity Frontal Crash Test. Annu. Proc. Assoc. Adv. Automot. Med..

[38] Fildes B., Keall M., Bos N., Lie A., Page Y., Pastor C., Tingvall C. (2015). Effectiveness of Low Speed Autonomous Emergency Braking in Real-World Rear-End Crashes. Accid. Anal. Prev..

[39] Ye X., Poplin G., Bose D., Forbes A., Hurwitz S., Shaw G., Crandall J. (2015). Analysis of Crash Parameters and Driver Characteristics Associated with Lower Limb Injury. Accid. Anal. Prev..

[40] Xu T., Sheng X., Zhang T., Liu H., Liang X., Ding A. (2018). Development and Validation of Dummies and Human Models Used in Crash Test. Appl. Bionics Biomech..

[41] Frye H.E., Ko D., Kotnik E., Zelt N. (2021). Policy Memo: Motor Vehicle Crash Testing Regulations for More Inclusive Populations. J. Sci. Policy Gov..

[42] Coufal T., Semela M. (2016). Determination of Selected Crash Parameters in Head-On Vehicle Collision with Rollover. Promet-Traffic Transp..

